# Connective tissue disease as a challenge in heart failure: Three case reports

**DOI:** 10.1097/MD.0000000000036885

**Published:** 2024-01-19

**Authors:** Ke Liu, Xuejiao Li, Dan Li

**Affiliations:** a Emergency Department of the Second Affiliated Hospital of ChongQing Medical University, Chongqing 400010, China; b Department of Cardiology, the Second Affiliated Hospital of ChongQing Medical University, Chongqing 400010, China.

**Keywords:** anti-synthetase syndrome, catastrophic antiphospholipid syndrome, connective tissue disease, heart failure, Takayasu arteritis

## Abstract

**Rationale:**

Connective tissue disease (CTD) is a heterogeneous group of chronic inflammatory autoimmune disorders derived from a systemically auto-immunological deregulation. CTD may affect cardiac structures through multiple pathophysiological mechanisms, and subclinical cardiac injury is common. Heart failure (HF) is one of the common complications in these patients.

**Patient concerns:**

Patients with CTD suffer an increased risk of cardiovascular disease and may have chest pain and shortness of breath.

**Diagnosis:**

HF is characterized by dyspnea or exertional limitation due to impaired ventricular filling and/or blood ejection. HF can be caused by other systemic diseases, not only by cardiovascular disorders but CTD. CTD may cause HF due to diffuse myocardial damage, heart valve damage, coronary ischemia, and so on.

**Interventions:**

The patient with catastrophic antiphospholipid syndrome take prednisone and warfarin. The patient with anti-synthetase syndrome was treated with immunoglobulin, followed by long-term oral medicines of prednisone, methotrexate, and folic acid.

**Outcomes:**

The symptoms of chest pain and shortness of breath for patients with CTD improved.

**Lessons:**

HF is one of the common complications in these patients with CTD, which has poor prognosis and severe aggravation. Once such patients experience chest pain, chest tightness, shortness of breath, etc, we should consider the possibility of HF. Early identification and correct treatment can delay the progression of HF, improve the prognosis, and enhance the quality of life for patients. Therefore, we should pay more attention to patients with CTD combined with HF.

## 1. Introduction

Connective tissue disease (CTD) is a heterogeneous rheumatic disease with the potential to have an impact on multiple body systems.^[1]^ Heart failure (HF) is a multifaceted syndrome that has a high mortality in the general population^[2]^ and is characterized by dyspnea or exertional limitation due to impaired ventricular filling and/or blood ejection. HF can be caused by other systemic diseases, not only by cardiovascular disorders but CTD. CTD may cause HF due to diffuse myocardial damage, heart valve damage, coronary ischemia, and so on.

## 2. Case presentations

### 2.1. Patient 1

A 20-year-old woman came to our department in November 2015 with severe dyspnea for 3 days. General condition was as followed: pulse rate 120 beats per minute, and oxygen saturation 93%. She suffered from paroxysmal nocturnal dyspnea and was unable to lie supine. Laboratory tests showed that brain natriuretic peptide concentration was 8451 pg/mL (N < 125 pg/mL), platelet count was 663 × 10^9^/L, erythrocyte sedimentation rate was 90 mm/h, D-dimer concentration was 2.3 mg/L; serum creatinine, aspartate transaminases, and alanine transaminases were negative. Electrocardiograph showed sinus tachycardia and positive T wave in V1 through V6 leads. Chest computed tomography (CT) revealed pulmonary embolisms of double inferior lung, and thrombus in the left ventricular aortic valve region and the apical region. Echocardiography demonstrated total cardiac enlargement, left ventricular hyperechoic thrombosis, pulmonary hypertension (51 mm Hg), and a reduced left ventricle ejection fraction (LVEF) of 21%. Lupus-like anticoagulant preliminary screening test (LA1)/lupus-like anticoagulant confirmatory test (LA2) = 1.36. The diagnosis of catastrophic antiphospholipid syndrome (CAPS) was made according to the criteria of Sydney International APS in 2006.^[3]^ The symptom of dyspnea was alleviated when the patient discharged. After that, she continued to take prednisone and warfarin for 1 month. Shortness of breath during exertion completely disappeared, and echocardiography showed EF increased to 58%, and left ventricular thrombosis disappeared.

### 2.2. Patient 2

A 81-year-old man was admitted to the hospital in July 2019 with chest pain and shortness of breath. Thirteen years ago, he underwent “aortic valve replacement” (biological valve). He was considered as “acute myocardial infarction” because of chest pain 2 years ago, then he received stent implantation and took medications regularly. Later, the patient repeatedly developed post-activity shortness of breath, paroxysmal nocturnal dyspnea, and orthopnea, accompanied by general fatigue. He was hospitalized for several times with troponin, creatine kinase, and creatine kinase isoenzyme positive. Echocardiography showed the status of aortic valve replacement, normal valve function, enlargement of the whole heart, pulmonary hypertension (moderate), and a reduced LVEF of 31%. Chest CT revealed interstitial changes in both lungs. No severe coronary artery stenosis was found on reexamination of coronary angiography. The skin on the radial and volar surfaces of the fingers was highly keratinized, rough, and pigmented. Symmetrical shedding and pigmentation were found in the joints of limbs and back. He was hunchback and had difficulty in lifting his head and bending his neck back. The tests of RO-52, anti-histone antibody, anti-PL-7 antibody, antinuclear antibody, and anti-M1-2α were all positive. No tumor was detected in the head, chest, and abdomen by CT examination. The diagnosis was considered as anti-synthetase syndrome (ASS), which involved myocardium, lung, and the proximal muscles. The patient was treated with immunoglobulin for 3 days, followed by long-term oral medicines of prednisone, methotrexate, and folic acid. Three months later, the symptoms of chest pain and shortness of breath improved slightly.

### 2.3. Patient 3

A 74-year-old man came to our hospital in March 2020 because of chest distress and shortness of breath. One year ago, the patient was diagnosed with acute cerebral infarction. Balloon dilation of the left subclavian artery and stent implantation of the right iliac artery were performed. Three months ago, he was hospitalized with chest distress and shortness of breath. Pulmonary artery CT angiography showed pulmonary embolism in the left upper lobe and right lower lobe. Echocardiography showed normal cardiac structure and ejection fraction. The difference of systolic blood pressure in both upper limbs was >10 mm Hg. The concentrations of erythrocyte sedimentation rate and interleukin-6 were increased significantly. The patient met 4 of the 6 criteria from the 1990 American College of Rheumatology classification^[4]^ and was diagnosed with Takayasu arteritis. However, he did not receive further examination and tocilizumab injection regularly for economic reasons. He suffered repeated shortness of breath with chest distress during follow-up. Reexamination of echocardiography showed enlargement of left atrium and left ventricle, decreased pulsation amplitude of the left ventricle posterior wall, and a reduced LVEF of 37%. Since then, he took the medicines for treatment of Takayasu arteritis and coronary heart disease regularly and felt much better than before.

## 3. Discussion

HF is a multifaceted syndrome that has a high mortality in the general population^[2]^ and is characterized by dyspnea or exertional limitation due to impaired ventricular filling and/or ejection of blood. The typical symptoms of HF include fatigue, dyspnea, orthopnea, paroxysmal nocturnal dyspnea, and ankle swelling; other symptoms may be presented include early satiety, bendopnea, and abdominal bloating.^[5]^ HF can be caused by other systemic diseases, not only by cardiovascular disorders, but also by CTD. CTD is a heterogeneous rheumatic disease that may affect multiple body systems.^[1]^

CAPS is a rare life-threatening antiphospholipid syndrome (APS) that can lead to multiorgan failure and high mortality.^[6]^ The etiology of APS remains unclear and may be related to genetic epitopes with infectious agents and other factors.^[7]^ Because of its vascularity, various organs and tissues are affected, including the cardiac system. The cardiac involvement of APS is multifactorial: thrombosis plays an important role in immune-mediated injury.^[8]^ Patient 1 had major clinical manifestations of HF and arterial thrombosis. In APS, antibodies in the myocardium can cause ventricular dysfunction.^[9]^ Coronary artery thrombosis and/or microvascular thrombosis may also cause APS-related ventricular dysfunction. Severe myocardial dysfunction may be a risk factor for left ventricular thrombosis. However, primary intracardiac thrombosis was found in other heart lumens, suggesting that antibodies may play a role in this process. Patient had an intracardiac thrombosis and pulmonary artery thrombosis, which also affect the oxygen supply to the heart and lungs and exacerbate HF. Patients with CAPS have a high mortality rate, so early detection and aggressive treatment are extremely important.

ASS is an autoimmune disorder characterized by interstitial lung disease, myositis, Raynaud phenomenon, fever, hyperkeratotic fingertips (mechanic’s hands), and arthritis, and is associated with antibodies to the aminoyl-tRNA synthase enzyme (the most common antibody being anti-JO-1).^[10]^ Other antibodies commonly associated with anti-synthase syndrome include anti-PL-7 and anti-PL-12.^[11]^ The anti-PL-7 antibody was found to be positive in patient 2. The clinical presentation and course of disease vary with the presence of anti-synthase antibodies.^[12]^ The role of anti-synthetase antibodies in the heart remains unclear. Kavita et al^[13]^ presented 2 cases of ASS associated with histologically proven myocarditis and clinical HF. Patient 2 had recurrent symptoms of chronic HF, interstitial lung disease, and the changes of skin and joints during the course of the disease. The mechanisms of ASS may be coronary artery inflammation, microvascular disease, intimal hyperplasia, or coronary vasospasm, all of which may result in impaired left ventricle function. There are no clear guidelines for the diagnosis of cardiac manifestations associated with ASS. Early and correct treatment can delay the progression of HF and improve the prognosis.

Takayasu arteritis is a rare granulomatous vasculitis characterized by granulomatous arteritis of the aorta and its branches.^[14]^ The aorta may be involved along its entire length. Takayasu arteritis also lead to in luminal narrowing and/or occlusion, organ ischemia, infarction, and functional failure, which are associated with considerable morbidity and premature mortality.^[14]^ In addition to the serious sequelae associated with cerebral, organ, and limb ischemia, patients may develop rapidly expanding aneurysms, pulmonary hypertension, or aortic rupture. Cardiac complications are common. Aortic insufficiency, cardiac ischemia, and myocardial infarction with HF are common causes of death.^[11]^ Shu et al^[15]^ reported a case of acute HF and nephrotic syndrome associated with Takayasu arteritis in a 16-year-old girl. As described above, patient 3 developed stenosis of left subclavian artery and lower limb artery followed by left and right pulmonary embolism, with normal cardiac structure and function. During the follow-up period, he did not take his medications regularly, and felt chest distress and shortness of breath. Echocardiography showed a decrease in ejection fraction, and coronary angiography showed severe coronary artery stenosis. If he follows the doctor’s instructions and adheres to the tocilizumab injection, his coronary arteries may not become severely narrowed for a period of time.

HF is a common disease in cardiovascular medicine. As a cardiovascular physician, when we encounter patients with symptoms of HF, we should consider not only cardiac disease, but also its pathogenesis, such as CTD, which may lead to HF as well. We should constantly improve the awareness of the disease and earlier diagnosis in clinical practice, treat different causes of HF, improve the quality of life of the patients, and prolong the life span.

## Author contributions

**Data curation:** Ke Liu, Xuejiao Li.

**Writing – original draft:** Ke Liu, Xuejiao Li,Dan Li.

**Writing – review & editing:** Ke Liu, Dan Li.
